# Hunger in Vulnerable Families in Southeastern Europe: Associations With Mental Health and Violence

**DOI:** 10.3389/fpubh.2020.00115

**Published:** 2020-04-15

**Authors:** Elena Jansen, Jamie M. Lachman, Nina Heinrichs, Judy Hutchings, Adriana Baban, Heather M. Foran

**Affiliations:** ^1^Institute of Psychology, Alps-Adria University, Klagenfurt am Woerthersee, Austria; ^2^Department of Social Policy and Intervention, University of Oxford, Oxford, United Kingdom; ^3^MRC/CSO Social and Public Health Sciences Unit, University of Glasgow, Glasgow, United Kingdom; ^4^Department of Psychology, University of Bremen, Bremen, Germany; ^5^School of Psychology, Bangor University, Wales, United Kingdom; ^6^Department of Psychology, Babeş-Bolyai University, Cluj-Napoca, Romania

**Keywords:** hunger, food insecurity, violence, mental health, support, socioeconomic status

## Abstract

**Background:** Hunger can influence healthy development of children and has been shown to be associated with other determinants of child health, such as violence within the family and maternal (mental) health problems. Whilst the majority of research has been conducted in high-income countries with vulnerable populations, less is known about the circumstances in low-and-middle-income countries. This study explored the experience of hunger in vulnerable families in three Southeastern European countries, and simultaneously examined relationships with four sets of risk factors—lack of financial, mental, familial, and social resources.

**Methods:** Families (*N* = 140) were recruited for a parenting intervention targeting child behavioral problems. Baseline data was collected on hunger, socioeconomic characteristics, mental health and wellbeing, family violence (i.e., child maltreatment and intimate partner violence), and social and emotional support. Univariate and multivariable risk factors of hunger were examined cross-sectionally with regression models.

**Results:** Overall, 31% of families experienced at least one form of hunger in the last month. Worse family functioning, current intimate partner violence, and more instances of child neglect showed univariate associations with family hunger. In hierarchical analysis, five risk factors remained significantly associated with the experience of hunger: lower adult educational, literacy level, emotional support, more children in the household and higher scores on parental depression, anxiety, and stress.

**Conclusions:** Hunger in Southeastern European families, among families with children showing elevated behavioral problems, was associated with more family violence, but specifically poorer mental health and less emotional support above and beyond socio-structural strains. Adapting parenting interventions to support the primary caregiver in getting more access to emotional support may potentially also change hunger and its association with health and violence. However, this hypothetical pathway of change needs explicit testing.

## Introduction

Undernutrition and hunger are affecting children around the world (1). Poor nutrition has detrimental effects on children's health, as well as on their physical, mental and social development (2–4). Consequences of undernutrition can include weakened immunity, susceptibility to long-term developmental delays or deficits, and increased risk of death, as well as prolonged negative effects on learning and economic performance (5–7). Undernutrition can result from illness, food shortage, inappropriate childcare or feeding practices, or a combination of these factors (5). Different causes of hunger may therefore be associated with different risk profiles. Having limited resources to purchase a variety of foods is often referred to as “food insecurity,” that is inadequate physical, social and economic access to nutritious and safe food (8).

Food insecurity and its potential consequence of experiencing hunger are two among many social and environmental determinants of child health. A range of these social and environmental factors have been found to cluster together, potentially creating chaotic living conditions for families (9) and reflecting a broader context of family adversity and trauma that can reinforce each other across generations (10, 11).

Food insecurity and the experience of hunger are closely linked to the socioeconomic status of the household (12). Food insecurity occurs alongside other forms of social inequality (13) and has therefore been interpreted as a proxy for poverty and economic hardship. Bocquire et al. (14) found relationships between food insecurity and adult age (younger), adult gender (women), family structure (single parents with children), and poorer material and housing conditions (non-home owners and lower income). Associations with indicators of family financial resources have further been reported in studies from Canada, England, Finland, France and the USA (15–19).

Shortage of food supply, and poverty in general, have been directly linked to poorer health of parents and children. Physical health problems occurring in residents of households with food insecurity include obesity/overweight due to overconsumption of low-cost, high-energy foods (20), hypertension and diabetes (21). Mental health problems include depression (13, 22, 23) and anxiety or psychological distress (24, 25). Families living with food insecurity also commonly experience feelings of shame, failure, desperation or being unfairly judged by others (25–28). Impacts on mental health, such as depression and wellbeing, limit the managerial capacity of caregivers (e.g., organizing and planning of food provision, motivation to shop and prepare food) (29, 30). This in turn can be overwhelming for parents and further influence the experience of food insecurity (e.g., feeling lack of control over the food environment) as well as impact the parent-child relationship by comprising parent-child attachment (31). Since mental health problems have also been shown to precede food insecurity and heighten its consequences (13, 32), it is unclear if hunger is indeed a causal risk factor for these adverse developmental outcomes for families or if hunger is rather a correlate, or a marker of these outcomes.

The abovementioned detrimental impacts of food insecurity can be particularly persistent if social resources of families are limited. Supportive relationships and social or instrumental support, either from within the family or outside, have been identified as buffers against the experience of adversity in food insecure households (33, 34). If a family member is additionally experiencing depression, s/he may have fewer supportive relationships (35) and an inability to connect with services that can assist with food hardship (29). Single female-headed households are at increased risk of experiencing food insecurity and poverty (12, 13, 30). Simultaneously, this group is also at increased risk of experiencing mental health problems and domestic violence (29).

Negative relationships, particularly in the form of family violence (i.e., child maltreatment and intimate partner violence), have been found more frequently in families residing in food insecure households or those affected by poverty. For instance, Jackson et al. (36) showed that child exposure to violence and/or victimization in the home early in life was almost six times higher in households experiencing persistent food insecurity (across three assessment waves) compared to food secure households. Similarly, plenty of evidence indicates that intimate partner violence (37–39) and hostile parenting (40) are prevalent in food insecure households. These circumstances suggest that familial resources are low. The consequences of victimization can easily impede the caregiver's capacity to meet family food needs since attention is mainly focused on safety and protecting the children (30). Several authors have highlighted the interplay amongst these risk factors, suggesting that food insecurity and family violence are driven by the same social and environmental risk factors (11, 29, 38, 39, 41), all of which can diminish mental health, especially if persistent (25, 42), and lead to more violent family interactions (43, 44).

While studies reporting the prevalence of hunger/food insecurity and family violence have focused on countries across the world, the majority of studies examining the relationships outlined above have mainly been conducted in the US and Europe. The focus has less frequently been on countries, for instance, in Southeastern Europe, many of which are classified as low-and middle-income countries (LMIC) (45). As such they experience higher rates of food insecurity, exposure to economic hardship, mental health issues, low levels of support and violence or victimization that can have far-reaching consequences on the health and development of children. These countries are more strained by economic hardship on a macro-contextual level and therefore offer a good opportunity to examine prevalence of hunger and associated risk profiles. Although these risk factors co-occur with the experience of hunger, most studies currently undertaken have not simultaneously examined relationships between these sets of risk factors but rather focused either on one or two sets only at the same time (such as mental health and family violence). Therefore, the aim of this study was to simultaneously examine associations between four sets of risk factors—lack of financial, mental, familial and social resources—and the experience of hunger in families living in three low-and middle-income Southeastern European countries. The primary goal was to identify those variables that are independently associated with and contribute incrementally to the phenomenon of hunger in LMICs.

## Methods

### Study Sample and Procedure

Data for the present study came from the pre-assessment of the feasibility phase of the RISE project, a multi-phase project with the overall goal of adapting, optimizing and testing a parenting intervention in three Southeastern European LMIC, using the Multiphase Optimization Strategy (MOST) and dimensions of the RE-AIM framework (46). Data for this first phase were simultaneously collected in North Macedonia, Republic of Moldova and Romania in 2018. The recruitment settings varied by country study site. Recruitment in Moldova was conducted in urban settings in Chisinau through youth-friendly health centers and at a local NGO targeting adults and youth with substance use disorder and HIV/AIDS. In Romania, recruitment was conducted in collaboration with educators in Cluj-Napoca and a community based organization in semi-rural village 80 miles from the city. Lastly, participants in North Macedonia were recruited in urban communities in Skopje at primary schools, kindergartens, family counseling services, and a community organization serving Roma families. Participants were recruited through flyers (e.g., via NGO's, kindergarten, schools); referrals by psychologists, social workers or teachers; social media pages of the local study institutes within each country; radio/TV advertisement; word-of-mouth by other parents and community leaders/ champions; door-to-door approaches; non-governmental and governmental organizations working with children and parents. Once potential study participants were connected to the study personnel, informed about the study and pre-screened for eligibility, an assessment was scheduled to gather consent and determine final eligibility. Participants needed to be aged 18 years or older, the primary caregiver of a child aged between 2 and 9 years, living in the same household as the target child for at least four nights a week and planning to do so during the course of the study, reporting elevated levels of behavior problems in the target child, agreeing to participate in the Parenting for Lifelong Health (PLH) for Young Children programme, and providing informed consent to participate in the study. Parents that either exhibited severe mental health problems or severe learning disabilities, or had been referred to child protection services due to child abuse were not eligible to participate in the study. Data collection took place either at participants' homes, the study institutes or any agreed-on location. Administration of consent forms and questionnaires was done with a Computer-Assisted Self-Interviewing (“CASI”) method with electronic-tablet technology. Trained data assessors read out questions if parents were unable to read, and assisted participants to type responses into the tablet if they were uncomfortable with or unable to use the tablet. In order to increase willingness to report stigmatizing experiences (47), audio-CASI was used, to administer sensitive items on the questionnaires (e.g., regarding child maltreatment/harsh parenting or intimate partner violence). RISE was approved by the human research ethics committee of the University of Klagenfurt and local ethics committees in North Macedonia, Moldova and Romania.

### Measurement Tools

All measurement tools that were not already available in the country-specific languages were translated and back-translated for the current project.

#### Demographic/Socioeconomic Factors

Participants reported on demographic and socioeconomic characteristics about themselves as well as their child. These included parent and child age and gender, parent education and literacy level, or number of children living in the household.

#### Experience of Hunger

Household hunger was assessed using three items on food shortage and hunger within the family that were based on the “Hunger Scale” (48–50). These items included: (1) “Do you ever run out of money to buy food for your home?”, (2) “Do you ever cut the size of meals or skip any meals because there is not enough food in the house?”, and (3) “Do you or any of your children go to bed hungry because there is not enough food to eat?”. Parents responded with yes or no. If parents confirmed the occurrence of hunger in the household, they were asked if it happened during the past 30 days, and subsequently, if it happened more than 5 times in the past 30 days. In the current study, individual incidences were examined and the overall prevalence was analyzed (i.e., experience of at least one form of hunger in the past 30 days).

#### Family Violence Factors

To assess harsh parenting, parents reported on 14 items which were based on the ISPCAN Child Abuse Screening Tool-Intervention scale (ICAST-I) (51, 52) and the Child Maltreatment Screener by Slep et al. (53). Four items assessed physical abuse (e.g., “In the past 4 weeks, how often did you discipline your child by slapping, spanking, or hitting with your hand?”), seven items assessed emotional abuse (e.g., “In the past 4 weeks, how often did you shout, yell or scream at your child?”) and three items assessed neglect (e.g., “How often in the past month did your child not get the food or drink that he/she needed even when there was money to pay for it?”). To assess the frequency of each behavior, the response scale ranged from 0 = “Never” to 8 = “8 or more times” in the past month. In this study, data for each of the three harsh parenting types were dichotomized to indicate the prevalence within the past month (i.e., previous harsh parenting or not).

Intimate partner violence was also assessed using questions from several sources. Participants reported on 15 victimizing and 14 perpetrating behaviors adapted from the Brief Screening Instrument for Partner Maltreatment by Heyman et al. (54) and the revised Conflict Tactics Scale (CTS2S) short form and full version (55, 56). Items in the current study referred to abuse within the last month (rather than the past year) and were assessed on a 9-point scale (0 = “Never happened” to 8 = “8 or more times” in the past month), with an additional response for incidents that happened in the past but not within the last month. Overall frequencies of victimization and perpetration (respective sum of items) were used as an indication of the level of severity, with possible ranges in the past month from 0 to 112 and 0 to 104. Due to non-normality, both variables were log transformed. Both measures of family violence were administered with an anonymous self-report response format. Parents who had trouble reading were able to enter their answers through audio-recorded instructions. This is the recommended method for data collection of sensitive problems to reduce underreporting (53, 54, 57).

#### Well-Being and Mental Health Factors

Parents provided information about their well-being and mental health via two widely used and validated measurement tools (58–60). The WHO-5 Well-Being Scale (61) measures parental psychological well-being on a 5-item scale. Parents indicated the well-being they experienced in the past month—original period was 2 weeks (e.g., “My daily life has been filled with things that interest me”) based on a 6-point Likert scale from 0 = “At no time” to 5 = “All of the time.” In the present study, the percentage score was used ranging from 0 to 100 (i.e., raw sum score multiplied by four) (62). Higher scores indicated better well-being. Internal reliability was acceptable (Cronbach's alpha = 0.77). Mental health was assessed using the Depression, Anxiety and Stress Scale (DASS, 21 items) (63). Participants reported on the frequency of symptoms in the previous week using a modified Likert scale (0 = “Never”, 1 = “Sometimes”, 2 = “Often”, 3 = “Always”; e.g., “I felt that I had nothing to look forward to”). The total DASS score ranged from 0 to 63 with higher scores indicating more psychological distress. Internal reliability was excellent (Cronbach's alpha = 0.91).

#### Social and Family Support Factors

The next set of parent-reported questions related to family functioning and emotional support. Parent perceived social support was measured using the emotional support subscale of the Medical Outcome Study Social Support Survey (MOS-SSS, eight items) (64). Parents reported how often they receive emotional support (e.g., “Someone you can count on to listen to when you need to talk”) using a 5-point Likert-like scale (1 = “None of the time” to 5 = “All of the time”). Higher mean scores indicated more emotional support. This scale has previously shown excellent test-retest reliability (α = 0.72–0.78) and internal consistency (α = 0.91–0.97) (65, 66), which was also found for the current study sample (α = 0.96). To assess family functioning, the general functioning subscale of the Family Assessment Device short form (FAD, 12 items) was used (67). Responses on each item (ranging from 1 = “Strongly agree” to 4 = “Strongly disagree”) were averaged after reverse coding where appropriate. Higher mean scores indicated more problems in family functioning. Previous studies have shown that the FAD is a valid instrument for assessing family outcomes in clinical trials with good internal consistency (68, 69). Similarly, in the current sample the internal consistency was good (α = 0.81).

### Data Analysis

Variables were inspected for non-normality and outliers. Where necessary, variables were either transformed or non-parametric tests were used. To ensure similarity of distributions across countries for merging the samples, differences were examined across the three countries, using ANOVAs for continuous variables and chi-square tests for categorical variables. To examine univariate relationships between the experience of hunger and the four sets of determinants (i.e., socioeconomic/demographic, family violence, mental health, social and family support), Independent sample *t*-tests, Mann Whitney tests and chi-square tests were performed depending on the scale of the measure. Significant variables from within each of the four sets of determinants were selected and then combined into a hierarchical logistic regression model to evaluate the change in variance explained. Variables were entered in the following order: Step 1—socioeconomic/demographic variables, Step 2—family violence related variables, Step 3—mental health-related variables, and Step 4—social and family support variables. Study country was entered as two dummy variables in Step 1 reflecting Moldova vs. Macedonia/Romania (dummy 1) and Romania vs. Macedonia/ Moldova (dummy 2), selecting Macedonia as reference category. Due to the high correlation between both intimate partner violence variables (*rho* = 0.75), models were run separately including victimization and perpetration, respectively, in Step 2. Results were the same and thus, only results with victimization are presented. Statistical significance was set at *p* < 0.05.

## Results

Of the 253 parents who were pre-screened, 162 were eligible (39 were ineligible and 52 could not be scheduled for an assessment). A further 22 parents were excluded at the final eligibility testing, leaving 140 eligible parents who consented and participated in the pre-assessment. Of those, 123 (87.9%) were currently in a relationship. Participant characteristics are presented in Table 1, also separated by country. Few country differences existed. The Romanian sample included a larger number of parents without a university or college degree and in line with this, a higher number of illiterate parents compared to the other two countries (both *p* < 0.001). Moldova showed more family dysfunction than Romania and less social support compared to the other two countries (both *p* < 0.05). Table 2 shows that 31% of the total sample experienced at least one form of hunger in the past 30 days. The proportion was highest in Romania (49%). In total, 19% experienced at least one form of hunger *more than 5 times* in the past 30 days and 6% experienced *all three forms of hunger* in the past 30 days. Table 3 shows the univariate relationships between the experience of hunger (i.e., at least one form of hunger in the past month) and all risk factors. Those that were significant were carried forward into the final hierarchical logistic regression model (see Table 4). Each step added significantly to the variance in the experience of hunger with the full models explaining 74% of the total variance. Three risk factors related to the socioeconomic/demographic, one to the mental health and one to the social and family support set, respectively. The odds of experiencing hunger were higher if the parent (i) had no university/college degree (OR = 13.79), (ii) could not, or could only read with difficulty (OR = 9.33), (iii) had more children living in their household (OR = 1.79), (iv) was more psychologically distressed (OR = 1.05), or (v) had less emotional support (OR = 0.22).

**Table 1:** Sample characteristics for the total sample (*N* = 140) and separated by country.

	**Total *n* (%) or *M* (SD)**	**Macedonia *n* (%) or *M* (SD)**	**Moldova *n* (%) or *M* (SD)**	**Romania *n* (%) or *M* (SD)**	***p*-values^b^**
Child age	5.8 (2.0)	5.7 (1.8)	6.3 (2.1)	5.5 (2.1)	0.169
Child gender (female)	77 (55.0)	26 (52.0)	26 (60.5)	25 (53.2)	0.683
Parent age	35.3 (7.5)	36.7 (4.3)	34.3 (7.5)	34.6 (9.8)	0.222
Parent gender (female)	137 (97.9)	47 (94.0)	43 (100)	47 (100)	0.063
Education level (no university/college)	68 (48.6)	18 (36.0)	15 (34.9)	35 (74.5)	<0.001
Literacy level (cannot/only read with difficulty)	32 (22.9)	6 (12.0)	4 (9.3)	22 (46.8)	<0.001
Number of children living in the household	2.3 (1.5)	2.2 (1.7)	1.9 (0.9)	2.6 (1.7)	0.109
Harsh parenting—physical (yes)	99 (70.7)	36 (72.0)	32 (74.4)	31 (66.0)	0.658
Harsh parenting—emotional (yes)	133 (95.0)	49 (98.0)	41 (95.3)	43 (91.5)	0.337
Harsh parenting—neglect (yes)	31 (22.1)	11 (22.0)	7 (16.3)	13 (27.7)	0.430
Intimate partner violence (IPV)—Victimization^a^	5.9 (11.5)	5.3 (8.4)	5.1 (9.1)	7.2 (15.5)	0.657
Intimate partner violence (IPV)—Perpetration^a^	4.6 (7.0)	4.0 (6.2)	3.6 (4.4)	6.2 (9.1)	0.192
WHO 5 well-being	53.1 (18.9)	50.3 (15.4)	54.9 (21.2)	54.3 (20.2)	0.442
DASS	31.0 (19.4)	29.8 (18.5)	32.6 (19.5)	30.8 (20.4)	0.789
FAD	1.9 (0.4)	1.9 (0.4)	2.1 (0.5)	1.8 (0.5)	0.007
MOS Social Support	3.6 (1.1)	4.0 (0.6)	3.0 (1.1)	3.8 (1.2)	<0.001

a *n = 123*.

b *Country differences were tested with ANOVA for continuous variables and chi-square test for categorical variables*.

*DASS, Depression Anxiety and Stress Scale (63); FAD, Family Assessment Device Short Form (67); MOS Social Support, Medical Outcome Study Social Support Survey (64)*.

**Table 2:** Experience of hunger within the total sample (*N* = 140) and separated by country.

	**Total *n* (%)**	**Macedonia *n* (%)**	**Moldova *n* (%)**	**Romania *n* (%)**
1. Run out of money to buy food—yes	51 (36)	11 (22)	15 (35)	25 (53)
2. Cut size or skip meal—yes	33 (24)	10 (20)	10 (23)	13 (28)
3. Child or parent go to bed hungry—yes	23 (16)	8 (16)	4 (9)	11 (23)
Experienced at least one form of hunger in past 30 days—yes	44 (31)^*^	9 (18)	12 (28)	23 (49)

* *Country differences in hunger were assessed with ANOVA and significant at p < 0.05*.

**Table 3:** Univariate relationships between the experience of hunger (i.e., at least one form of hunger in the past month) and risk factors (*N* = 140).

	**Experience of hunger Yes *M* ± SD or count**	**Experience of hunger No *M* ± SD or count**	***p*-value**	**OR**
Child age	6.1 ± 2.0	5.7 ± 2.0	0.302	
Parent age	33.8 ± 8.2	36.0 ± 7.2	0.108	
Number of children living in the household	3.0 ± 1.7	1.9 ± 1.3	<0.001	
WHO 5 well-being	45.9 ± 18.1	56.3 ± 18.5	0.002	
DASS	42.0 ± 19.6	26.0 ± 17.1	<0.001	
FAD	2.1 ± 0.5	1.8 ± 0.5	0.008	
MOS Social Support	3.1 ± 1.2	3.9 ± 0.9	<0.001	
IPV—Victimization^a^	10.7 ± 16.9	3.7 ± 6.9	0.004	
IPV—Perpetration^a^	8.5 ± 10.4	2.8 ± 3.5	0.012	
Child gender			0.770	0.9
Female	25	52		
Male	19	44		
Education level			<0.001	13.0
Less than university/college	39	29		
University/college	5	67		
Literacy level			<0.001	21.5
Cannot/only read with difficulty	26	6		
Can read easily	18	90		
Harsh parenting—physical			0.213	0.6
No previous abuse	16	25		
Previous abuse	28	71		
Harsh parenting—emotional			0.316^*^	2.9
No previous abuse	1	6		
Previous abuse	43	90		
Harsh parenting—neglect			0.006	3.1
No previous abuse	28	81		
Previous abuse	16	15		

* *For one or more cells the expected count is <5*.

a *n = 123 because not all parents were in a relationship*.

*OR, Odds ratio; DASS, Depression Anxiety and Stress Scale (63); FAD, Family Assessment Device Short Form (67); MOS Social Support, Medical Outcome Study Social Support Survey (64); IPV, intimate partner violence*.

*Country differences were tested with Independent Samples T-test for normally distributed continuous variables, Mann-Whitney test for non-normally distributed continuous variables, and chi-square test for categorical variables*.

**Table 4:** Hierarchical logistic regression for the experience of hunger (i.e., at least one form of hunger in the past month) and four sets of risk factors.

**Variables^a^**	**B**	**Wχ^**2**^**	**OR**	**95% CI**
Step 1—socioeconomic/demographic	Nagelkerke *R*^2^ = 0.58			
Education level (university)	2.63	9.31^*^	13.79	2.56–74.44
Literacy level (can read)	2.23	5.80^*^	9.33	1.52–57.39
Number children in household	0.58	6.20^*^	1.79	1.13–2.82
Country dummy1 (Moldova vs. rest)	−0.39	0.16	0.68	0.10–4.71
Country dummy2 (Romania vs. rest)	−1.58	2.78	0.21	0.03–1.32
Step 2—family violence	Nagelkerke *R*^2^ = 0.64			
Child neglect (yes)	−1.21	1.74	0.30	0.05-1.80
IPV—Victimization^b^ (log transformed)	1.14	1.58	3.11	0.53–18.29
Step 3—mental health	Nagelkerke *R*^2^ = 0.68			
DASS total score	0.05	4.21^*^	1.05	1.00–1.10
WHO total score	−0.01	0.35	0.99	0.94–1.03
Step 4—social support	Nagelkerke *R*^2^ = 0.74 Goodness of fit χ^2^ (df) = 92.45 (11)^*^			
FAD total score	−0.14	0.02	0.87	0.12–6.52
Emotional support	−1.52	7.37^*^	0.22	0.07–0.66

*B, unstandardized regression coefficient; Wχ^2^, Wald χ^2^-test; OR, Odds ratio; 95% CI, 95% CI for Odds ratio; IPV, intimate partner violence; DASS, Depression Anxiety and Stress Scale (63); FAD, Family Assessment Device Short Form (67); MOS Social Support, Medical Outcome Study Social Support Survey (64)*.

* *p < 0.05*.

a *Values presented are taken from final model*.

b *Analyses were run with IPV perpetration and all variables that are statistically significant in the current model, remained statistically significant with IPV perpetration as an independent variable rather than victimization*.

## Discussion

The aim of this study was to explore the reported levels of hunger in families with elevated levels of child behavioral problems living in three Southeastern European countries as well as to examine associations between four sets of risk factors and the experience of hunger. Risk factors were divided into socioeconomic/demographic, family violence, mental health-related, and social or family support factors. One third of the sample experienced at least one form of hunger in the past month, with differences apparent between countries. The majority of the risk factors explored showed a univariate association with the experience of hunger and together explained more than 70% of the variance. In the final model, five risk factors remained significant, particularly highlighting the role of socioeconomic/demographic risks compared to all other sets of risk factors.

Using the Food Insecurity Experience Scale (70) across 28 European Union countries (71), a prevalence of 18% of households experiencing a moderate (4%) to severe (14%) inability to access food has been reported. The prevalence in the European Commonwealth of Independent States identifying former parts of the Soviet Union (including Moldova) was similar with 17% of households reporting moderate (2%) to severe (15%) inability to access food. While the rate seen in the current sample was higher (31%), this is probably related to the difference in the recruitment and thus vulnerability of the selected sample, but also due to the measurement tools used (e.g., food insecurity measure vs. experience of hunger measure). Due to the lower income and higher poverty rates in comparison to even the newer European member states, food insecurity has been reported to be a concern for a large proportion of the population in Romania (72) and Moldova (73, 74), and likely also in North Macedonia since it is also a LMIC.

Three socioeconomic/demographic factors were associated with the experience of hunger. This is in line with previous studies. Smith et al. (33) found that three of the five characteristics that were associated with the largest increase in the likelihood of experiencing food insecurity around the world included low education levels, low household income, and being unemployed. The other two included less social capital and weak social networks which also align with findings of the current study and are discussed below. Economic/financial resources, or hardship on the other end of the continuum, are well known buffers, or risk factors. Extended exposure to poverty, especially during early childhood, has adverse impacts, with hunger being one of them (75, 76). Families living in circumstances of socioeconomic disadvantage are not only more likely to reside in food insecure households but also experience other, overlapping family adversities, such as family violence (29), mental health difficulties (25, 77), substance abuse (78, 79), and reported child behavioral problems (13).

Although intimate partner violence (both victimization and perpetration) and child neglect, but not physical or emotional abuse, showed a univariate association with the experience of hunger, none of the family violence factors remained significant once entered into the hierarchical regression model. Other studies have found consistent relationships, for instance, between food insecurity and domestic violence (OR = 2.36, 95% CI: 1.18–4.73) (13) or children witnessing physical violence in the home (moderate-to-severe food insecurity, OR = 2.66, 95% CI: 2.26–3.09) (41). Notably, many of the previous studies highlighting relationships between food insecurity and family violence did not simultaneously examine the range of risk factors that were explored in the current study. This may be one reason why the strength of association with family violence factors was diluted, once other risk factors were taken into consideration. This does not indicate that domestic violence is not a relevant risk factor, but suggests that the relationship between domestic violence and food insecurity may be mediated by other risk factors.

In the current study, a higher score on the Depression, Anxiety and Stress scale was associated with slightly higher odds of experiencing hunger (OR = 1.05, 95% CI 1.00–1.10) whilst a higher well-being score only showed a trend in lower odds of experiencing hunger (OR = 0.99, 95% CI 0.94–1.03). This is in line with a range of previous studies presenting a close link between food insecurity and poorer mental health and specific psychological stressors. According to Jones et al. (80), these associations are found across the globe, independent of socioeconomic status. Also drawing on the 2014 Gallup World Poll (81), Frongillo et al. (82) indicated that food insecurity was strongly negatively associated with subjective well-being as well when measured in a large global sample (above 120,000 participants from 138 countries). We only found a small association which was no longer significant when other sets of risk factors were simultaneously included. At present, a bidirectional relationship is accepted in that food insecurity can both reflect but also engender cascading mental health challenges (41).

Finally, social and family support factors were examined. Interestingly, in the current study no association was seen between general family functioning (“In times of crisis we can turn to each other for support” or “We are able to make decisions about how to solve problems”) and the experience of hunger, whilst a significant association was found for emotional support. Although the latter measure includes questions such as “Someone to give you good advice about a crisis” or “Someone to confide in or talk to about yourself or your problems” which could also refer to family members, this measure of support potentially captures support from the wider social network. Associations between food insecurity and weaker social networks (i.e., dissatisfied with ability to make friends) or less social capital (i.e., cannot count on friends and family in times of need) were found using data including 134 countries (33). Tsai et al. (34), whose measure assessed emotional or instrument support provided by a family member (i.e., intimate partner, mother or father), found that instrumental social support functioned as a buffer against adverse impacts of food insecurity. These findings suggest that the effect might be related to the type of support received, rather than the person or proximity from which support is provided.

Findings of the current study have to be considered in the light of possible limitations. The current sample may not be representative of the average North Macedonian, Moldovan and Romanian parent since participants were selected for a parenting intervention focused on child behavioral problems and were reporting child behavioral problems as a recruitment criterion. In addition to the interplay of risk factors mentioned above, families reporting child behavioral problems may also be more likely to experience all the other family adversities (13). The current sample may therefore have been more likely to report experiencing hunger. Due to the cross-sectional nature of the data no statement could be made about the direction of relationships. The current data were sourced from the feasibility pilot phase of RISE (46). Consequently, analyses were exploratory, the sample size was small, limiting the ability to conduct sub-sample analyses and further group comparisons, and the assessment of the experience of hunger was not the main focus of the study with only three items included for measurement. However, other studies, such as Bocquier et al. (14) used a single-item to assess food insecurity (“Which of the following statements best described the food currently consumed in respondents' household?”), and our items are very similar to three of the seven used by Wehler et al. (30) from the Community Childhood Hunger Identification Project. Advantages include capturing the perspective of parents in three Southeastern European countries that have not been specifically targeted for this research focus before and the fact that several risk factor sets were assessed simultaneously with concurrent measurement of all variables. Finally, there is potential bias of underreporting relating to self-reported measurement of family violence. In order to minimize social desirability bias or underreporting, audio-CASI (i.e., computer assisted self-interviewing) was used in the present study, allowing participants (even if illiterate) to respond to questions without the assistance of the interviewer. Further, prevalence rates found in the current sample are similar to other studies of intimate partner violence in the past year [e.g., 10.1% (83) or 18.3% (84) compared to 11.5% in this sample].

Findings from our study indicate that the risk factors related to the experience of hunger in the three Southeastern European countries North Macedonia, Republic of Moldova and Romania are similar to those reported for other countries. Given increasing evidence in the literature that the abovementioned factors are intertwined, examination of this complex interplay by modeling more comprehensive relationships (e.g., through path modeling) seems warranted. Further investigations utilizing tools that are comparable to other studies and including larger and more representative samples will additionally advance our understanding of commonality and representativeness of findings obtained from less frequently studied populations. If comparable, it should be considered whether or not attempts to simultaneously address a range of family adversities (food insecurity, psychological well-being, family violence), for instance through providing intimate partner violence and mental health resources, as has been done via the Women, Infants, Children (WIC) Nutrition Program in the United States (29), are also useful in other countries or are unsuitable due to cultural and systemic differences. Additionally, testing the benefit of pairing parenting interventions with economic strengthening programmes targeted at food insecurity may be particularly useful for LMIC. The current results relating to emotional support are promising because controlling for a number of other types of risk, emotional support appeared to be an important variable in this cross-sectional interplay. Further investigation about change in emotional support following participation in an intervention program and its impact on hunger seem warranted, particularly since group-based programs, such as PLH which is applied in the RISE study, offer the potential to generate social support (85).

## Data Availability Statement

The datasets generated for this study will not be made publicly available according to the EU funder approved data management plan. Data from the RISE study will only be made publicly accessible at the end of the study period and after publication of the related study results. Requests to access these datasets should be directed to Professor Heather Foran, heather.foran@aau.at.

## Ethics Statement

The studies involving human participants were reviewed and approved by the human research ethics committee of the University of Klagenfurt, the local ethics committees in North Macedonia, Moldova and Romania. The patients/participants provided their written informed consent to participate in this study.

## Author Contributions

AB, HF, NH, JH, and JL contributed to the conception and design of the study. EJ managed the data, performed the statistical analysis, and wrote the first draft of the manuscript. All authors contributed to manuscript revision, read, and approved the submitted version.

### Conflict of Interest

JL and JH are co-developers of PLH for Young Children, which is licensed under a Creative Commons 4.0 Non-commercial No Derivatives license, and one of the co-founders of the Parenting for Lifelong Health initiative, receive occasional fees for providing training and supervision to facilitators and coaches, and have participated (and are participating) in a number of research studies involving the program, as an investigator, and the University of Oxford, and Bangor University receive research funding for these. JL is also the Executive Director of Clowns Without Borders South Africa, a non-profit institution responsible for the dissemination of the program in Africa and East Africa. Likewise, JH is the director of the Children's Early Intervention Trust, a non-profit institution responsible for the dissemination of the program in Europe and led the evaluation of the program in a pre-post trial in Montenegro and contributed to the trial in the Philippines. NH serves as an international advisory board member for the Triple P program. The remaining authors declare that the research was conducted in the absence of any commercial or financial relationships that could be construed as a potential conflict of interest.
