# Corrigendum: Hunger in Vulnerable Families in Southeastern Europe: Associations With Mental Health and Violence

**DOI:** 10.3389/fpubh.2020.00336

**Published:** 2020-07-29

**Authors:** Elena Jansen, Jamie M. Lachman, Nina Heinrichs, Judy Hutchings, Adriana Baban, Heather M. Foran

**Affiliations:** ^1^Institute of Psychology, Alps-Adria University, Klagenfurt am Woerthersee, Austria; ^2^Department of Social Policy and Intervention, University of Oxford, Oxford, United Kingdom; ^3^MRC/CSO Social and Public Health Sciences Unit, University of Glasgow, Glasgow, United Kingdom; ^4^Department of Psychology, University of Bremen, Bremen, Germany; ^5^School of Psychology, Bangor University, Wales, United Kingdom; ^6^Department of Psychology, Babeş-Bolyai University, Cluj-Napoca, Romania

**Keywords:** hunger, food insecurity, violence, mental health, support, socioeconomic status

In the original article, we neglected to include the funder Faculty of Humanities at the Alps-Adria University Klagenfurt, to Elena Jansen covering the article processing charges. The corrected **Funding** statement appears below.

The authors apologize for this error and state that this does not change the scientific conclusions of the article in any way. The original article has been updated.

